# Assessment of Perception on Careers in Preventive Medicine Among Medical Students From Taif University: A Cross-Sectional Study

**DOI:** 10.7759/cureus.91361

**Published:** 2025-08-31

**Authors:** Ammar Saud Altowairqi, Abdullah M Alqethami, Muhammad Shakir Raza, Muhammad Taimoor Raza, Shaima Ali Alghamdi, Abdullah Ali Alsharif, Rawan Khalid Asali, Abdulaziz Abdulmalik Althubyani, Ghala Saud Alosaimi

**Affiliations:** 1 Department of Public Health, Saudi Ministry of Health, Taif, SAU; 2 Department of Cardiology, Rai Medical College Teaching Hospital, Sargodha, PAK; 3 Department of Psychology, University of Sargodha, Sargodha, PAK; 4 Department of Medicine and Surgery, Taif University, Taif, SAU

**Keywords:** career choice, medical students, preventive medicine, saudi arabia, specialty selection

## Abstract

Objectives

Preventive Medicine (PM) remains underrepresented despite its importance in public health, particularly in Saudi Arabia. This study aimed to assess advanced clinical students' (Years 5-6) perceptions of PM, examine factors associated with PM perceptions, and evaluate perceived barriers to PM selection at Taif University.

Methods

An analytical cross-sectional study surveyed Year 5 and Year 6 medical students (n=245). The questionnaire measured demographic information, specialty decision-making factors, perceptions of PM, and COVID-19 pandemic influences. Internal consistency was evaluated via Cronbach's alpha. Descriptive statistics, t-tests, correlations, and regression analyses were conducted, with significance at p<0.05.

Results

Of 245 advanced clinical students surveyed (113 Year 5; 132 Year 6), only seven (2.9%) indicated intention to pursue PM. Reliability measures for key scales were as follows: Specialty Confidence Index (α=0.55, interpreted cautiously), Factors Influencing Specialty Choice (α=0.80), and Perceptions of Preventive Medicine (α=0.68). Higher specialty confidence (β=0.35; p<0.001) and career factors (β=0.22; p<0.001) predicted favorable PM perceptions but not actual PM selection. The logistic regression model for PM choice was non-significant (p=0.189), likely due to the small PM group (n=7).

Conclusions

Despite recognizing PM's public health value, very few students chose it as their specialty. This workforce gap necessitates curriculum reform, mentorship development, and systemic changes to PM's professional standing. Study limitations include the unvalidated questionnaire and single-institution design.

## Introduction

Medical students' specialty choices significantly impact the distribution of the healthcare workforce [1,2], yet Preventive Medicine (PM) remains underrepresented despite its importance in public health [3,4]. In Saudi Arabia, where non-communicable diseases account for 73% of all deaths [5] and the Health Sector Transformation Program under Vision 2030 prioritizes the prevention of health risks [6], the PM workforce remains small relative to population needs [7].

PM specialists in Saudi Arabia focus on disease surveillance, health policy development, outbreak investigation, and population health management at both regional and national levels [8]. Working primarily through public health directorates, the Saudi Center for Disease Control and Prevention, and academic institutions [9], these specialists, also termed Community Medicine practitioners in some institutions, apply epidemiology, biostatistics, environmental health, and health promotion to prevent disease rather than treat individual patients [10].

Entry to the PM specialization requires completing six years of medical school and a one-year internship, followed by competitive selection through the Saudi Commission for Health Specialties (SCFHS) standardized examinations and interviews for the four-year residency program [11,12]. While specific workforce data remains unpublished, the PM specialist pool is acknowledged as insufficient for population needs, a pattern observed across Gulf countries [13,14]. This data absence itself indicates the specialty's marginalization and justifies research into factors affecting PM career selection [7,14].

Multiple factors are associated with specialty decisions, including perceptions of prestige, financial considerations, and educational exposure [15-17]. Limited clinical exposure and mentorship opportunities can misrepresent PM's scope [18,19], while financial concerns may overshadow altruistic motivations [20,21]. The COVID-19 pandemic highlighted PM's critical role in disease surveillance and prevention [22], particularly relevant for Saudi Arabia, which hosts millions for Hajj and Umrah annually, requiring robust public health infrastructure for mass gathering health management [23,24].

Despite PM's alignment with Saudi Vision 2030 initiatives [25,26], little is known about Saudi medical students' perceptions of PM careers. Previous international studies have documented PM selection rates of 1-3% [27,28], but Saudi-specific data remain absent. This study, conducted at a major public medical school in western Saudi Arabia, assessed the perceptions of advanced clinical students (Years 5 and 6) regarding PM careers.

The primary objective of the study was to assess the proportion of advanced clinical students expressing interest in pursuing PM as a postgraduate specialty. The secondary objectives were to examine factors associated with PM perceptions and identify perceived barriers to PM selection.

This study hypothesizes that social, educational, and financial factors are significantly associated with perceptions of PM among medical students, that structural and perceptual barriers deter specialty selection despite recognition of PM's public health value, and that COVID-19 experiences have influenced students' PM career considerations.

## Materials and methods

Study design and setting

This analytical cross‑sectional study evaluated advanced clinical students' (Year 5 and Year 6) perceptions of PM and examined factors associated with interest in PM at Taif University College of Medicine in western Saudi Arabia. The college follows a six‑year curriculum (basic sciences in Years 1-3 and clinical training in Years 4-6), with Year 6 constituting the final year. We included both Year 5 and Year 6 because students in these adjacent stages have completed most core clinical rotations and are actively considering postgraduate specialty choices. As a single‑institution study, external generalizability may be limited beyond similar settings. Measurement considerations (including internal consistency and the absence of external validation for the de novo questionnaire) are detailed in Instrumentation and revisited in Limitations.

Participants and sampling

We targeted advanced clinical students (Year 5 and Year 6) enrolled during the 2024-2025 academic year at Taif University College of Medicine. In the Saudi six‑year curriculum, Year 6 constitutes the final year; Year 5 students have completed most core clinical rotations and are actively forming postgraduate specialty preferences. Including both adjacent years allowed analysis across contiguous stages of specialty decision‑making.

We used a census approach: all enrolled Year 5 and Year 6 students listed by the registrar were invited (n=260) via institutional channels. Year and sex were used only for descriptive stratification in analyses; no probabilistic sampling was performed. Eligibility criteria were enrollment in Year 5 or Year 6 and provision of electronic informed consent. Exclusion criteria were incomplete questionnaires on pre‑specified mandatory items and suspected duplicate submissions based on data quality screening. A total of 245 students provided complete responses (response rate 94.2%), comprising 113 Year 5 and 132 Year 6 participants, with 119 males (48.6%), 123 females (50.2%), and three undisclosed (1.2%).

Sample size planning

For the planned multiple linear regression with up to 12 predictors, conventional assumptions of a medium effect size (f²=0.15; α=0.05; and power=0.80) indicate a minimum required sample of n≈127 (G*Power 3.1, Heinrich-Heine-Universität Düsseldorf, Düsseldorf, Germany). Our analyzed sample (n=245) exceeded this threshold. Because data collection was anonymous, non-responder characteristics could not be assessed; we note potential non-response bias and single-site generalizability as limitations. Study procedures adhered to the Saudi National Committee of Bioethics guidelines [29].

Ethical approval and consent

The study protocol received approval from the Institutional Review Board (IRB) of the Directorate of Health Affairs-Taif (IRB registration number with KACST, KSA: HAP-02-T-067; approval number: 833; date: 18/07/2023), in accordance with the Declaration of Helsinki guidelines [30].

Instrumentation

A semi-structured, self-administered questionnaire was developed de novo for this study. Content domains were informed by medical specialty choice literature [15,31,32], though all items were originally authored for the Saudi context. Internal consistency (Cronbach's α) was assessed for multi-item scales; formal content validity, construct validity, and test-retest reliability were not conducted.

Development steps

Item generation was guided by (i) factors commonly cited in specialty decision‑making (e.g., lifestyle, income, prestige, mentorship, program length), (ii) perceptions specific to PM, and (iii) potential COVID‑19 influences on attitudes toward PM given the Kingdom's mass gathering context (Hajj/Umrah) and the heightened visibility of public health during the pandemic. A small pilot for clarity (face validity check) was performed before the main data collection; no separate formal pre‑test was undertaken. Because the pilot was for wording/clarity only, pilot responses were not included in the main analyses. The final questionnaire appears in the Appendices.​​​​​​​

Questionnaire components

The questionnaire was comprised of the following components: (a) demographics: age, gender, academic year, grade point average (GPA) (4.0 scale), residence (urban/rural), and physicians in the immediate family (parents, siblings, spouses); (b) specialty decision status: three categories, that is, "Decided" (firm choice), "Uncertain" (considering options), and "Not decided" (no preference); (c) Specialty Confidence Index (SCI; 5 items; 1-5 Likert): five items assessing decision certainty (α=0.554; interpreted cautiously due to modest reliability); (d) Factors Influencing Specialty Choice (FISC; 12 items; 1-5 Likert): 12 items covering career factors rated 1-5 (α=0.800); (e) Perceptions of Preventive Medicine (PM Perceptions; 8 items; 1-5 Likert with "N/A/Don't know" option): eight items with 5-point Likert and "N/A" option (α=0.683) (N/A responses were treated as missing; scales were computed when ≥70% items were answered); (f) COVID-19 Influences and Experiences (CIE; 6 items; 1-5 Likert): six items examining pandemic impact on specialty perceptions (α=0.814), operationalizing our hypothesis about COVID-19's influence on PM interest; (g) PM knowledge: two items testing prevention concepts and one familiarity rating; and (h) open-ended questions: optional items on PM advantages/disadvantages (exploratory use only). ​​​​​​​Missing data were handled via mean imputation for scales with ≥70% completion; otherwise, listwise deletion. The complete questionnaire appears in the Appendices.

Data collection procedure

The questionnaires were administered electronically over a four-week period in November 2024 using Google Forms (Google LLC, Mountain View, California, United States). Responses were collected anonymously, with access restricted to the researchers responsible for analysis. The survey link was circulated via the institution's official email lists for Year 5 and Year 6 cohorts; it was not publicly posted. To preserve confidentiality, login authentication was not required. We reviewed the response file for obvious duplicates (identical timestamps and full response sets) and found no apparent duplications. As authentication was not enforced, the possibility of ineligible responses cannot be ruled out and is addressed in the Limitations.

Data management and statistical analysis

All valid questionnaires were coded and entered into IBM SPSS Statistics for Windows, Version 27.0 (IBM Corp., Armonk, New York, United States). Descriptive statistics summarized demographic variables, specialty preferences, and scale scores. Internal consistency of multi-item scales was evaluated using Cronbach's alpha coefficients. Inferential analyses included independent-sample t-tests, one-way ANOVA, Pearson correlations, multiple linear regression, and logistic regression. Statistical significance was set at p<0.05 (two-tailed).

## Results

Participant characteristics

A total of 245 advanced clinical students (Years 5-6) participated (response rate=94.2%). The sample included 119 males (48.6%), 123 females (50.2%), and three undisclosed (1.2%). The mean age was 23.03 years (SD=1.02; range: 18-26). Year distribution was as follows: Year 5: 113 (46.1%) and Year 6: 132 (53.9%). GPA (4.0 scale) categories were as follows: <2.5: 102 (41.6%), 2.5-2.99: 19 (7.8%), 3.0-3.49: 78 (31.8%), and 3.5-4.0: 46 (18.8%). Most participants (88.2%) resided in urban areas.

Thirty-two percent (80/245) reported having an immediate family member who is a physician (defined in Methods). Regarding specialty decision status, 111 (45.3%) reported being decided, 105 (42.9%) uncertain, and 29 (11.8%) not decided. Among the 111 decided students, the most common planned specialties were Internal Medicine (n=50; 45% of decided) and Family Medicine (n=42; 37.8% of decided). Seven students (6.3% of decided; 2.9% of the total sample) indicated PM.

For missing/N/A handling, 15 participants selected "N/A/Don't know" on one or more PM items; these responses were treated as missing for scale scoring. Unless otherwise stated, N=245 with pairwise deletion applied for analyses.

Reliability and descriptive statistics of key measures

Table 1 summarizes the internal consistency and central tendency for the four multi‑item scales. The FISC scale showed good reliability (α=0.800). The PM Perceptions scale showed acceptable reliability (α=0.683), and the CIE scale showed high reliability (α=0.814). The SCI showed modest internal consistency (α=0.554); therefore, all SCI‑related findings should be interpreted with caution.

**Table 1:** Descriptive statistics of key scales Interpret SCI results with caution, given α=0.554. FISC: Factors Influencing Specialty Choice; SCI: Specialty Confidence Index; PM Perceptions: Perceptions of Preventive Medicine; CIE: COVID-19 Influences and Experiences; SD: standard deviation

Scale	Items	Score range (min-max)	Cronbach's α	Mean±SD
FISC	FISC1-FISC12	12-60	0.800	42.22±8.09
SCI	SCI1-SCI5	5-25	0.554	17.67±3.68
PM Perceptions	PM1-PM8	8-40	0.683	29.17±5.03
CIE	CIE1-CIE6	6-30	0.814	17.22±5.31

Knowledge and perceptions of PM

As shown in Table 2, 31.4% correctly identified primary prevention, and 33.9% correctly identified the Saudi Center for Disease Prevention and Control as the principal entity for national preventive programs. These findings indicate notable knowledge gaps in core PM concepts.

**Table 2:** Knowledge of Preventive Medicine concepts (n=245)

Knowledge item	Correct response n (%)
Identify "primary prevention" correctly	77 (31.4%)
Identify the principal national entity for preventive programs (Saudi Center for Disease Control and Prevention)	83 (33.9%)

Correlations between key measures

Pearson correlations are shown in Table 3. SCI correlated positively with FISC (r=0.270; p<0.001) and Perceptions of PM (r=0.420; p<0.001). FISC also correlated with Perceptions of PM (r=0.334; p<0.001). CIE showed weak but statistically significant correlations with the other scales (r=0.178-0.199; all p<0.01). As a guide, coefficients <0.30 are considered weak and may have limited practical significance.

**Table 3:** Correlations between study variables (n=245) Statistical test: Pearson r; **p<0.01. Values <0.30 denote weak correlations; SCI findings should be read with caution, given α=0.554.

Variable	1	2	3	4
Specialty confidence	-	-	-	-
Factors influencing specialty	0.270**	-	-	-
Perceptions of Preventive Medicine	0.420**	0.334**	-	-
COVID-19 influences and experiences	0.178**	0.199**	0.194**	-

Group comparisons

Academic Year (Year 5 vs. Year 6)

Compared with Year 5, Year 6 students placed more weight on specialty‑influencing factors (FISC mean difference=3.18; p=0.002) and held more positive perceptions of PM (mean difference=1.94; p=0.002) (Table 4). SCI means did not differ significantly (p=0.185). No differences emerged for CIE (p=0.565).

**Table 4:** Comparison of mean scores between Year 5 and Year 6 students Statistical test: two‑tailed tests; *p<0.05. Interpret SCI results with caution, given α=0.554. FISC: Factors Influencing Specialty Choice; SCI: Specialty Confidence Index; PM Perceptions: Perceptions of Preventive Medicine; CIE: COVID-19 Influences and Experiences

Scale	Year 5 (n=113)	Year 6 (n=132)	t(243)	P-value
FISC	40.50±7.96	43.68±7.93	-3.120	0.002*
SCI	17.34±3.96	17.96±3.41	-1.329	0.185
PM Perceptions	28.12±5.64	30.06±4.26	-3.055	0.002*
CIE	17.44±5.10	17.04±5.48	0.577	0.565

Place of Residence (Rural vs. Urban)

No statistically significant differences were observed by residence. A trend suggested stronger weighting of career‑related influences among urban students (p=0.077) (Table 5). 

**Table 5:** Comparative analysis by place of residence (urban vs. rural) (n=245) Statistical test: independent‑sample t‑tests (two‑tailed). FISC: Factors Influencing Specialty Choice; CIE: COVID-19 Influences and Experiences

Scale	Urban (n=216) mean±SD	Rural (n=29) mean±SD	P‑value
FISC	42.55±8.08	39.72±7.83	0.077
CIE	17.07±5.12	18.28±6.54	0.252

Physicians in the Immediate Family

Students without a physician in their immediate family reported higher specialty confidence (18.10±3.40 vs. 16.80±4.09; p=0.009; d≈0.36), with no differences on FISC or PM Perceptions (Table 6) (immediate family is defined in Methods).

**Table 6:** Comparative analysis by family background (physician in the immediate family vs. none; n=245) Statistical test: independent‑sample t‑tests (two‑tailed); *p<0.05. Interpret SCI results with caution, given α=0.554. SCI: Specialty Confidence Index; FISC: Factors Influencing Specialty Choice; PM Perceptions: Perceptions of Preventive Medicine

Scale	Physician in the family (n=80) mean±SD	No physician in the family (n=165) mean±SD	t(243)	P‑value
SCI	16.80±4.09	18.10±3.40	−2.617	0.009*
FISC	41.85±8.26	42.39±8.02	−0.496	0.621
PM Perceptions	28.79±5.21	29.35±4.96	−0.639	0.524

Decision to Pursue PM

Only seven students (2.9%) selected PM. Relative to peers, these students had lower specialty confidence (14.43±3.31 vs. 17.77±3.65; p=0.018; d≈0.91) and lower FISC scores (34.29±8.62 vs. 42.45±7.97; p=0.008). Given the very small PM group (n=7), these comparisons are exploratory and should be interpreted cautiously (SCI findings also warrant caution, given α=0.554).

Logistic regression: determinants of choosing PM

A logistic regression model (outcome: choosing PM vs. other specialties) did not reach overall significance (χ²(15, N=238)=19.56; p=0.189), consistent with limited power given the small PM group (n=7). Familiarity with PM showed a significant coefficient (B=−1.115; p=0.023; Wald=5.152), indicating that higher self‑rated familiarity (with the present coding) was associated with lower odds of choosing PM (Table 7). This counterintuitive pattern should be viewed as hypothesis‑generating only.

**Table 7:** Logistic regression predicting the likelihood of choosing Preventive Medicine (n=238) Model fit: χ²(15, N=238)=19.56; p=0.189 (non-significant overall model). The Wald statistic tests whether individual coefficients differ from zero; for 1 degree of freedom, Wald values exceeding 3.84 indicate p<0.05. Results should be interpreted cautiously due to the small number of students choosing PM (n=7) and modest reliability of the Specialty Confidence Index (α=0.554). *p<0.05

Predictor	B	SE	Wald	P-value	Odds ratio (95% CI)
Factors influencing specialty	0.076	0.052	2.134	0.144	1.079 (0.974-1.194)
Specialty confidence	0.153	0.125	1.503	0.220	1.166 (0.913-1.488)
Preventive Medicine familiarity	-1.115	0.491	5.152	0.023*	0.328 (0.125-0.858)
Constant	2.736	2.451	1.246	0.264	15.427

Multivariate linear regression: predicting perceptions of PM

A linear regression predicting perceptions of PM explained ~29% of variance (R²=0.291; adjusted R²=0.254; F(12,225)=7.71; p<0.001). Higher specialty confidence (B=0.480; p<0.001) and greater weighting of specialty‑influencing factors (B=0.146; p<0.001) were associated with more favorable PM perceptions, whereas having a physician in the immediate family was associated with slightly lower perceptions (B=−1.358; p=0.032) (Table 8). These associations should be interpreted within the measurement limits (SCI α=0.554) and cross‑sectional design.

**Table 8:** Multiple regression predicting perceptions of Preventive Medicine (n=238) R²=0.291; adjusted R²=0.254; F(12,225)=7.71; p<0.001, where F represents the F-statistic testing overall model significance with 12 predictors and 225 residual degrees of freedom. Statistical test: multiple linear regression; *p<0.05. Interpret SCI results with caution, given α=0.554. B: unstandardized coefficient; SE: standard error; β: standardized coefficient indicating the relative strength of association when variables are on comparable scales

Predictor	B	SE	β	P-value
Specialty confidence	0.480	0.086	0.351	<0.001*
Factors influencing specialty	0.146	0.041	0.234	<0.001*
Having a physician in the family	-1.358	0.628	-0.126	0.032*

## Discussion

This analytical cross-sectional study of 245 advanced clinical students at a Saudi medical school revealed that only 2.9% intend to pursue PM, despite moderate-to-positive perceptions of the specialty. Year 6 students demonstrated more favorable PM attitudes and stronger consideration of career factors compared to Year 5 students. While higher specialty confidence and career factor weighting correlated with positive PM perceptions, these did not translate into specialty selection. Knowledge assessment revealed fundamental gaps, with only 31.4% correctly identifying primary prevention concepts.

Interpretation in the context of prior literature

The 2.9% PM selection rate aligns with international patterns documenting 1-3% selection rates [27,28] yet remains concerning given Saudi Arabia's preventive health priorities [6]. This paradox, recognizing PM's importance while avoiding it professionally, has been documented globally and reflects persistent challenges in recruitment.

The observed knowledge deficit regarding primary prevention echoes findings from other medical education contexts where prevention concepts receive insufficient emphasis [33]. This misunderstanding may perpetuate the perception that PM lacks clinical substance compared to intervention-focused specialties [34]. Studies from comparable Middle Eastern contexts report similar patterns, where procedural specialties maintain prestige hierarchies despite health system needs for prevention expertise [35].

Year-based differences suggesting evolving perceptions align with longitudinal research demonstrating that clinical exposure shapes specialty preferences [4,36]. However, the modest effect sizes and cross-sectional design preclude causal attribution. The negative association between physician family members and PM perceptions corroborates research suggesting medical families often steer students toward traditionally prestigious specialties [37,38].

The counterintuitive finding that PM familiarity predicted lower selection likelihood warrants careful interpretation. Similar paradoxes have been reported where increased exposure to undervalued specialties reinforces negative perceptions rather than correcting them [39]. This may reflect accurate awareness of PM's lower compensation and prestige rather than measurement error [40].

COVID-19 context and missed opportunities

Despite the pandemic highlighting public health's critical role, COVID-19 influences showed only weak correlations with PM perceptions (r<0.2). This finding contradicts expectations that pandemic experiences would enhance PM appeal [41] and suggests that crisis visibility alone cannot overcome structural deterrents [42]. Studies from other pandemic-affected regions similarly report minimal shifts in public health career interest despite increased awareness [43].

Implications for medical education

The knowledge gaps identified necessitate curriculum reform beyond adding PM content. Successful models from other contexts suggest integrating population health longitudinally rather than in isolated blocks [44,45]. 

Early Integration

Institutions successfully increasing PM interest embed prevention concepts from Year 1, using clinical cases to demonstrate population impact [46]. Problem-based learning, incorporating outbreak investigations and screening program evaluations, has shown promise [47].

Mentorship Programs

Structured mentorship increases specialty commitment across underrepresented fields [48]. PM-specific mentorship programs combining research opportunities with policy engagement have demonstrated success in other settings [49].

Assessment Reform

Aligning examinations with prevention competencies signals institutional prioritization [50]. Objective Structured Clinical Examination (OSCE) stations incorporating population health scenarios can reinforce PM's clinical relevance [51].

Policy and workforce implications

The disconnect between Vision 2030's preventive health emphasis and student career intentions reflects a critical misalignment between workforce needs and current student intentions [6]. International evidence suggests that financial incentives alone rarely overcome prestige deficits [52]; comprehensive approaches addressing compensation, career progression, and professional recognition prove more effective [53].

Saudi Arabia's unique position hosting mass gatherings (Hajj/Umrah) creates specific PM workforce needs [23,24]. Leveraging this distinctive context through specialized training tracks could differentiate Saudi PM programs while addressing national priorities [54]. Countries successfully expanding PM workforces have combined targeted recruitment, enhanced training pathways, and visible career progression [55].

Methodological considerations and limitations

While achieving high response rates and capturing contiguous training years strengthen internal validity, several limitations constrain interpretations.

Firstly, the unvalidated instrument represents the study's primary weakness. Without established psychometric properties, measurement error may substantially affect findings. The SCI's modest reliability (α=0.554) particularly limits confidence in associations involving specialty confidence. Future research must prioritize validated instruments or undertake rigorous validation processes [56].

Secondly, the single-institution design limits generalizability across Saudi medical schools with varying curricula and regional contexts [57]. The cross-sectional design precludes causal inference, a critical limitation when examining factors "influencing" career decisions [58].

Lastly, the small PM-interested group (n=7) creates statistical instability, particularly in regression models. This reflects the underlying problem, namely, PM's unpopularity, but limits analytical power [59]. Anonymous data collection without authentication, while protecting confidentiality, introduces potential response contamination [60].

Strengths and contributions

Despite limitations, to our knowledge, this is among the first reports focused specifically on PM career intentions among Saudi medical students, addressing a critical knowledge gap. The high response rate (94.2%) and comprehensive assessment across multiple constructs offer valuable baseline data. Transparent reporting of reliability coefficients and model diagnostics enables appropriate interpretation.

Future research directions

Longitudinal cohort studies tracking students from admission through residency selection would establish temporal relationships between exposures and career decisions [61]. Multi-institutional studies could identify curriculum features associated with higher PM interest [62]. Qualitative research exploring prestige perceptions and career decision-making processes in the Saudi context remains essential [63].

Intervention studies testing targeted strategies, that is, mentorship programs, longitudinal PM tracks, and financial incentives, should employ rigorous evaluation designs [64]. International comparative research could identify transferable strategies from countries successfully expanding PM workforces [65].

## Conclusions

This study reveals a significant paradox: despite intellectual appreciation of PM, very few Saudi final-year medical students choose it as a specialty. This mismatch stems from entrenched prestige hierarchies, financial concerns, limited mentorship, and historically undervalued public health roles. However, opportunities exist to enhance PM's appeal, particularly with heightened visibility post-COVID-19 and alignment with Vision 2030 healthcare priorities.

## Appendices

Questionnaire: perceptions of Preventive Medicine careers among Saudi medical students

Introduction

Dear Participant,

You are invited to participate in a research study aimed at understanding the perceptions of final-year medical students toward careers in Preventive Medicine and the factors influencing their choice of postgraduate specialty. Your responses will contribute to improving medical education and career guidance in Saudi Arabia.

This questionnaire will take approximately 15 minutes to complete. Your participation is voluntary and confidential. You may skip any question or withdraw at any time without consequences.

Section A: Demographic Information

Age: _____ years

Gender:

☐ Male ☐ Female ☐ Prefer not to say

Academic Year:

☐ Fifth Year ☐ Sixth Year

Grade Point Average (GPA):

☐ Below 2.5 ☐ 2.5-2.99 ☐ 3.0-3.49 ☐ 3.5-4.0 ☐ Above 4.0

Place of Residence:

☐ Urban ☐ Rural

Do you have any physicians in your immediate family?

☐ Yes ☐ No

Section B: Specialty Preference and Indecision

Have you decided on your postgraduate specialty?

☐ Yes ☐ No ☐ Unsure

If Yes, which specialty are you considering? (Select one)

☐ Internal Medicine ☐ Surgery ☐ Pediatrics ☐ Obstetrics and Gynecology ☐ Family Medicine ☐ Preventive Medicine ☐ Other (please specify): _______________

Please rate your agreement with the following statements: (Scale: 1: Strongly disagree; 2: Disagree; 3: Neutral; 4: Agree; 5: Strongly agree)

SCI1. I feel confident about my specialty choice 1 2 3 4 5

SCI2. I worry that I might not be satisfied with my specialty choice 1 2 3 4 5

SCI3. I find it difficult to choose between different specialties 1 2 3 4 5

SCI4. I feel pressure to decide on a specialty quickly 1 2 3 4 5

SCI5. I wish I had more information about different specialties 1 2 3 4 5

Section C: Factors Influencing Specialty Choice

How influential are the following factors in your specialty choice? (Scale: 1: Not at all influential; 2: Slightly influential; 3: Moderately influential; 4: Very influential; 5: Extremely influential)

FISC1. Personal interest in the field 1 2 3 4 5

FISC2. Anticipated income 1 2 3 4 5

FISC3. Work-life balance 1 2 3 4 5

FISC4. Length of residency training 1 2 3 4 5

FISC5. Prestige of the specialty 1 2 3 4 5

FISC6. Influence of mentors or role models 1 2 3 4 5

FISC7. Job market opportunities 1 2 3 4 5

FISC8. Opportunity to help others 1 2 3 4 5

FISC9. Intellectual challenge 1 2 3 4 5

FISC10. Alignment with religious or cultural values 1 2 3 4 5

FISC11. Impact of Vision 2030 on healthcare 1 2 3 4 5

FISC12. Technological advancements in the field 1 2 3 4 5

Are there any other factors not listed above that influence your specialty choice? Please explain:

Section D: Knowledge and Perceptions of Preventive Medicine

How familiar are you with the specialty of Preventive Medicine?

☐ Not at all familiar ☐ Slightly familiar ☐ Moderately familiar ☐ Very familiar ☐ Extremely familiar

Please answer the following questions about Preventive Medicine:

a. Which of the following is NOT a core area of Preventive Medicine?

☐ Epidemiology ☐ Biostatistics ☐ Surgery ☐ Health Policy

b. Which level of prevention focuses on early detection of diseases?

☐ Primary prevention ☐ Secondary prevention ☐ Tertiary prevention ☐ Quaternary prevention

c. In Saudi Arabia, which government body is primarily responsible for preventive health programs?

☐ Ministry of Health ☐ Saudi Center for Disease Prevention and Control ☐ Saudi Food and Drug Authority ☐ All of the above

Please indicate your level of agreement with the following statements: (Scale: 1: Strongly disagree; 2: Disagree; 3: Neutral; 4: Agree; 5: Strongly agree; N/A: Not applicable/don't know)

PM1. Preventive Medicine is crucial for improving population health 1 2 3 4 5 N/A

PM2. Preventive Medicine offers a good work-life balance 1 2 3 4 5 N/A

PM3. Careers in Preventive Medicine are financially rewarding 1 2 3 4 5 N/A

PM4. Preventive Medicine lacks prestige compared to other specialties 1 2 3 4 5 N/A

PM5. Preventive Medicine aligns well with Saudi Arabia's healthcare goals 1 2 3 4 5 N/A

PM6. Preventive Medicine doesn't involve enough patient interaction 1 2 3 4 5 N/A

PM7. Technological advancements will increase the importance of Preventive Medicine 1 2 3 4 5 N/A

PM8. I would consider a career in Preventive Medicine if I knew more about it 1 2 3 4 5 N/A

What do you perceive as the main advantages of a career in Preventive Medicine?

What do you perceive as the main disadvantages of a career in Preventive Medicine?

Section E: Influences and Experiences

How has the COVID-19 pandemic influenced your perception of Preventive Medicine?

☐ Much more positive ☐ Somewhat more positive ☐ No change ☐ Somewhat more negative ☐ Much more negative

How influential are the following in shaping your perception of medical specialties? (Scale: 1: Not at all influential; 2: Slightly influential; 3: Moderately influential; 4: Very influential; 5: Extremely influential)

CIE1. Medical school curriculum 1 2 3 4 5

CIE2. Clinical rotations 1 2 3 4 5

CIE3. Media portrayals (TV shows, movies, etc.) 1 2 3 4 5

CIE4. Social media 1 2 3 4 5

CIE5. Family opinions 1 2 3 4 5

CIE6. Peers' opinions 1 2 3 4 5

Please describe a specific experience or encounter that has significantly shaped your perception of Preventive Medicine (positively or negatively):

Section F: Future Perspectives

How do you think the role of Preventive Medicine in Saudi Arabia will change in the next 10 years?

☐ Become much more important ☐ Become somewhat more important ☐ Stay about the same ☐ Become somewhat less important ☐ Become much less important

What do you think could be done to make Preventive Medicine a more attractive specialty choice for medical students in Saudi Arabia?

Conclusion

Thank you for completing this questionnaire. Your responses will contribute to our understanding of medical students' perceptions of Preventive Medicine and help inform future educational and policy decisions in Saudi Arabia.

If you have any questions or concerns about this study, please contact [Researcher's Name] at [contact information].
